# Delineating reclamation zones for site-specific reclamation of saline-sodic soils in Dushak, Turkmenistan

**DOI:** 10.1371/journal.pone.0256355

**Published:** 2021-08-17

**Authors:** Elif Günal

**Affiliations:** Department of Soil Science and Plant Nutrition, Tokat Gaziosmanpaşa University, Tokat, Turkey; Mendel University of Agriculture and Forestry: Mendelova univerzita v Brne, CZECH REPUBLIC

## Abstract

Soil salinization is the widespread problem seriously affecting the agricultural sustainability and causing income losses in arid regions. The major objective of the study was to quantify and map the spatial variability of soil salinity and sodicity. Determining salinity and sodicity variability in different soil layers was the second objective. Finally, proposing an approach for delineating different salinity and sodicity zones was the third objective. The study was carried out in 871.1 ha farmland in Southeast of Dushak town of Ahal Province, Turkmenistan. Soil properties, including electrical conductivity (EC), soil reaction (pH), sodium adsorption ratio (SAR), calcium carbonate and particle size distribution (clay, silt and sand fractions) in 0–30, 30–60, 60–90 and 90–120 cm soil layers were recorded. The EC values in different soil layers indicated serious soil salinization problem in the study area. The mean EC values in 0–90 cm depth were high (8 dS m^-1^), classifying the soils as moderate to strongly saline. Spatial dependence calculated by the nugget to sill ratio indicated a strong spatial autocorrelation. The elevation was the primary factor affecting spatial variation of soil salinity in the study area. The reclamation of the field can be planned based on three distinct areas, i.e., high (≥12 dS m^-1^), moderate (12–8 dS m^-1^) and low (<8 dS m^-1^) EC values. The spatial trend analyses of SAR values revealed similar patterns for EC and pH; both of which gradually decreased from north to the south-west. The amount of water needed to leach down the salts from 60 cm of soil profile is between 56.4–150.0 ton ha^-1^ and the average leaching water was 89.8 tons ha^-1^. The application of leaching water based on the amount of average leaching water will result in higher or lower leaching water application to most locations and the efficiency of the reclamation efforts will be low. Similar results were recorded for sulfur, sulfuric acid and gypsum requirements to remediate sodicity. The results concluded that the best management strategy in planning land development and reclamation schemes for saline and sodic soils require accurate information about the spatial distribution of salinity and sodicity across the target area.

## Introduction

Soil salinity is the most widespread soil degradation processes mainly in the arid and semiarid regions of the world. Soil salinity in agricultural field is created due to higher evapotranspiration rates compared to precipitation, characteristics of soil and topography that impede water drainage and cause salt accumulation in the soil profile [1]. Salinity restricts production of arable lands, causing degradation globally of 0.3–1.5 million hectare year^−1^ [2] and >50% of arable lands of the world may be affected by the salt accumulation until 2050 [3,4].

Salt and sodium-affected lands should be used in agricultural production to meet the demands of increasing global population for food and fiber [5]. Therefore, productivity function of salt and sodium-affected soils should be improved to extend the coverage area of arable lands. However, reclamation of saline and saline-sodic lands using chemicals have become expensive with the increasing use in industry [6]; therefore, optimization is needed with respect to inputs and time [7,8]. Assessing salinity and sodicity distribution is a prerequisite to identify the problem areas and develop appropriate management practices [9].

Reclamation of saline sodic soils involves the release of calcium either from the added calcium amendments or from the native calcium carbonate, which could be mobilized through addition of acids or acid formers [10], exchange with the sodium adsorbed on exchange complexes of soils, improvement of soil physical structure, and lowering the pH value. Therefore, in saline-sodic soils, sodium concentration have to be reduced by using chemical amendments, such as sulfur, sulfuric acid and gypsum. Afterwards, soluble salts need to be leached from the root zone through irrigation. If pH is high and soil contains sufficient calcium carbonate, then compounds such as sulfur or sulfuric acid help to increase the dissolution of calcite in calcareous saline-sodic soils and release calcium to soil solution [1,11].

Generally, amendments such as gypsum, elemental sulfur or sulfuric acid used in reclamation are determined based on the calculation of mean values of electrical conductivity (EC) or sodium adsorption ratio (SAR) values of the field ignoring the spatial distribution along the field [12]. Application of chemicals and leaching water based on the mean values of the salinity parameters reduce reclamation success and increase input cost due to high spatial variability of salinity in the area [8]. In addition, application of freshwater for leaching has great limitation in arid regions due to the limited resources and expansion of agricultural lands. Site-specific reclamation practices considering the variability of salinity are needed to maximize the benefits from remediation efforts. Therefore, quantifying spatial variability in soil salinity or sodicity is necessary to increase reclamation efficiency. Determining spatial distribution and mapping soil salinity are preliminary steps towards decision making to reclaim salt and sodium-accumulated areas for the adaptation of appropriate management practices [13–15]. Site-specific input application management zones is adapted to increase nutrient use efficiency and crop yield, improve profitability by decreasing the costs, and reduce environmental impacts [16]. Similarly, Shaddad et al. [14] showed the effectiveness of geostatistical tools in accurately assessing spatial variability of soil variables deemed relevant for reclamation of saline soils and nitrogen fertilization.

The information on spatial distribution of soil salinity has great importance for planning soil reclamation [17]. Variability of salinity in a field can accurately be characterized using geostatistics, which reduces the number of samples to be collected and analyzed [13]. Plenty of studies have been carried out on spatial variability of soil properties, including salinity; however, few studies have considered the spatial structure and variability of soil salinity and sodicity to guide the site-specific remediation [18,19]. Understanding the spatial variability of soil salinity and sodicity is important for designing site-specific sustainable management decisions for salinity and sodicity reclamation.

Agriculture is the source of income for many of countries in Central Asia. However, soil salinity, erosion and desertification in Aral Basin are the major land degradation processes. Saline soils in Turkmenistan occupy ~95.8% of the irrigated lands, which has a strong impact on plant establishment, land revegetation and performance of land productivity in arid zone [20]. The contribution of agriculture to the GDP in Turkmenistan is 7.5% [21]. The ecological conditions around the Aral Sea have been degraded due to the extensive furrow irrigation. Excessive use of water, extracted from the Amudarya and Syrdarya rivers, elevated groundwater tables and insufficient drainage have led to the salinization of agricultural fields [20]. Reclaiming saline soils in the region is necessary for agricultural production. Reclamation of salt affected lands is expected to improve ecological environment and stimulate rural development. Leaching of salts is the common practice to reclaim saline soils in the region [22]. However, water supply in Turkmenistan is a major problem and have been exacerbated recently by geopolitical developments [23]. The main purpose of this study was to quantify and map the spatial variability of soil salinity and sodicity in South-East of Dushak town of Ahal Province in Turkmenistan. Determining the distribution of salinity and sodicity in different soil layers was the second major objective of the study. Reclaiming different salinity and sodicity zones with the application of varying rates ıf chemicals and leaching was the final objective of the study. It was hypothesized that the field will show significant spatial variability in salinity and sodicity. The spatial variability would require varying rates of chemicals and leaching water in the field. The results will help in effective reclamation and would assist in reducing the costs associated with chemicals and water application.

## Materials and methods

### Study area

**Fig 1:**
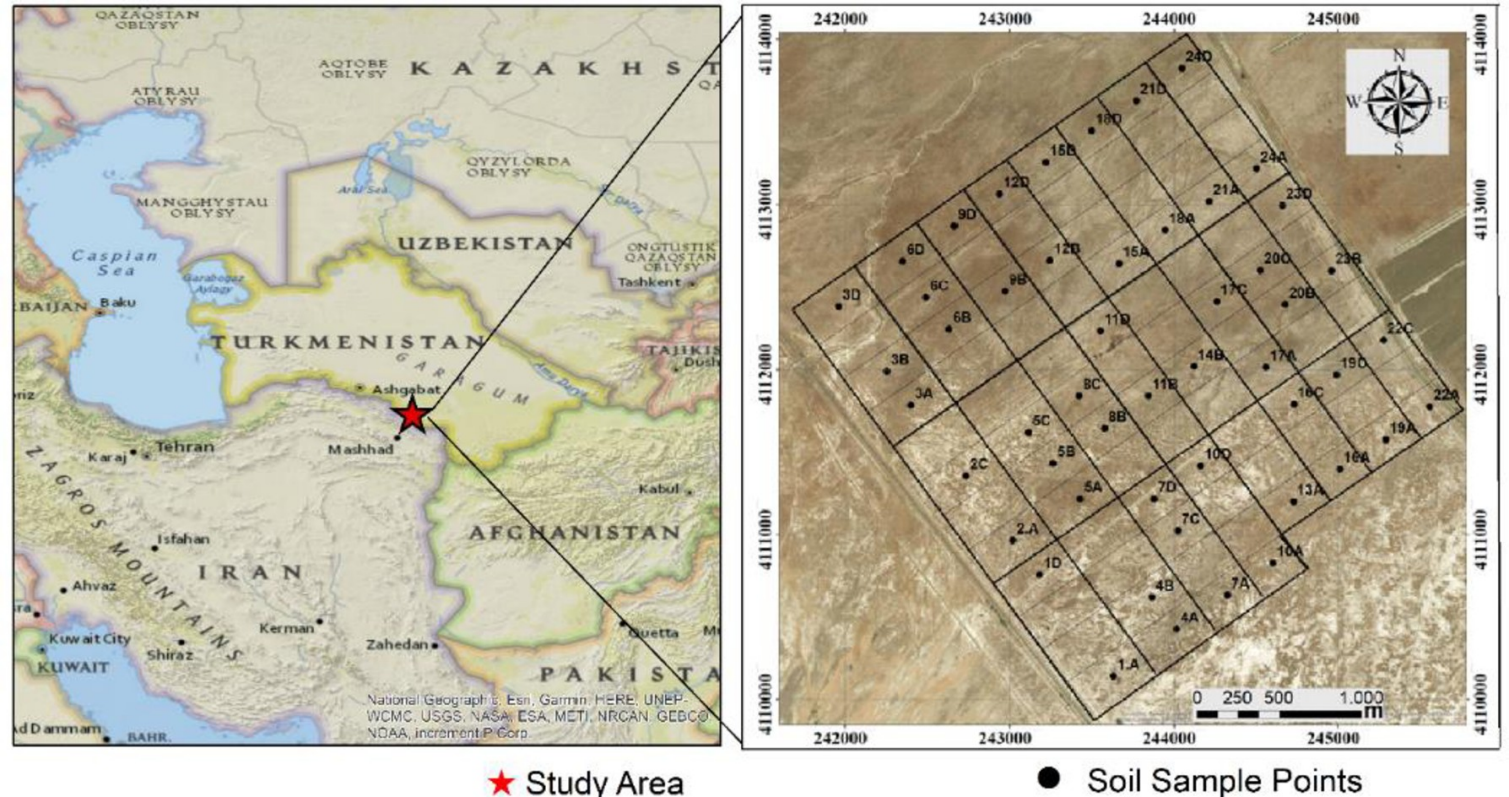
Location of study area and distribution of the sampling locations.

The study area is located between 37°5’59’’ - 37°7’18’’ north and 60°6’48’’ - 60°7’8’’east longtitudes in the Southeast of Dushak town, Ahal Province, Turkmenistan (Fig 1). The study area is spread over 871 ha. Agricultural production is mostly practiced in Tejen region of the country. Higher annual evaporation compared to the precipitation, and saline groundwater [24] caused an increase in salt content of soils in the area.

The climate is continental and arid (a desert climate) with hot summers and cold winters [25]. The study area receives little rainfall throughout the year. Annual precipitation is 189 mm and climate is classified as BWk according to Köppen and Geiger. The least amount of precipitation occurs in July (0 mm), while long term average the highest precipitation occurs in March with an average of 41 mm. The months with no or the lowest precipitation have the highest temperatures. The average annual temperature is 15.7°C. The hottest month of the year is July with an average of 29.0°C, while January has the lowest average temperature of the year (2.2°C). The average annual evaporation from water surface varies from 2000 to 2300 mm [26]. Since the annual potential evapotranspiration is higher than the annual rainfall, irrigation is required to cultivate crops [20]. The average long-term climatic data of the experimental site is summarized in Table 1.

**Table 1:** Averages of long-term climate data of the Dushak city, Turkmenistan [26].

Attributes/Months	1	2	3	4	5	6	7	8	9	10	11	12
**Mean Temperature (°C)**	2.2	4	8.9	15.3	21.6	26.2	29	27.1	22.7	16.7	9.8	5.1
**Min. Temperature (°C)**	-3.3	-1.4	2.9	8.6	14.3	18.2	21.1	18.8	14.2	8.8	3.0	0.0
**Max. Temperature (°C)**	7.7	9.4	14.9	22.1	29	34.2	36.9	35.5	31.2	24.6	16.7	10.2
**Precipitation (mm)**	25	22	41	33	20	3	0	0	2	9	15	19

The topography of the study area is flat with altitude ranging from 257 (south) to 240 m (north) and from 248 (east) to 240 m (west). Due to flat topography, the ground water flow is limited, resulting in a shallow groundwater depth which induced the accumulation of salts in soil profile. The soils in the study area are classified as Fluvisols in Soil Taxonomy [27] and gleyic calcaric Arenosols in World Reference Base [28].

### Soil sampling

The study area was divided into 330 × 330 m regular grids and the locations of the grid nodes were recorded prior to the sampling and uploaded to a Global Positioning System (GPS) receiver. At each grid node, composite soil samples were collected at approximately corners of fifty locations from four layers at a depth increment of 30 cm. Soil samples were analyzed for particle size distribution (clay, silt and sand contents), pH, electrical conductivity (EC), sodium adsorption ratio (SAR) and calcium carbonate (CaCO_3_) contents.

### Soil analysis

Electrical conductivity and pH were measured in saturation paste using a pH-meter and a combined glass electrode [29]. Organic matter was analyzed using the Walkley Black method [30], and calcium carbonate content was determined with a calcimeter [31]. Particle size distribution of soil samples was determined using hydrometer method [32]. All analyses were repeated twice.

### Calculation of soil amendments for reclamation

The amount of chemicals was calculated using the guidelines explained in Kanber and Ünlü [33] as follows (Eq 1).


$$\mathrm{CR}=(EW*10^{-5})(A*Ds*Bd)(\frac{{ESP}_{i}-{ESP}_{f}}{100})CEC$$ Eq 1

In the equation; CR is the amount of chemicals needed for reclamation (ton ha^-1^). EW is the equivalent weight (for sulfur EW is 16, for sulfuric acid EW is 48.96 and for gypsum EW is 86). A is the coverage area of the field (m^2^), Ds is the depth of soil profile to be reclaimed (m), Bd is the soil bulk density (ton m^-3^) and ESP_i_ is the initial exchangeable sodium percentage (ESP, %). ESP_f_ is the final ESP value (%), which was set to 6%, and CEC is the cation exchange capacity of soil (me 100 g soil^-1^), which was used as 20 me 100 g soil^-1^.

The ESP value was calculated using the equation given in Anaplı [34] as follows (Eq 2).


ESP=((100×(0.0198+0.015×SAR))|(1+(0.0198+0.015×SAR) Eq 2

The soil pores must first be saturated with water to wash and remove salts from the soil profile to the desired depth (60 or 120 cm). The amount of water required for saturation of soil pores was calculated using the Eq 3, and total amount of leaching water needed was calculated according to the Reeve’s [35] equation (Eq 4).


dw=((Wfc−Wa)|10)×(BdxD) Eq 3

Where, the dw is the amount of needs for the saturation of soil pores (cm), Wfc is the water content at field capacity, Wa is the available water content, Bd is the soil bulk density (ton m^-3^), and D is the depth (cm).

The amount of water needed to leach salts from soil profile depends on the initial salinity, desirable salinity and the soil depth on which leaching will be accomplished.


$$\frac{Dlw}{Ds}=\frac{1}{5(\frac{C}{C_{0}})}+0.15$$ Eq 4

Where, the Dlw: Leaching water requirement (cm), Ds is the leaching depth (cm), C_f_ is the final EC value, and C_o_ (dS m^-1^) is the initial EC value (dS m^-1^). In this study, the C_f_ value was set to 2 dS m^−1^ which is considered safe for most cultivated crops [36].

Bulk density for each sampling point and depth was estimated using pedotransfer functions using soil texture (clay and sand content) and organic matter content [37] as follows (Eq 5);

p=100/((X|Po)+{(100−X)|Pm}) Eq 5

On the equation, p is bulk density (ton m^-3^), X is organic matter content (%), Po is mean bulk density of organic matter, and Pm is bulk density of mineral fraction, which ranges between 1 and 17 ton m^-3^ depending on sand and clay content [37].

### Spatial analysis

The main tool of geostatistics to reveal spatial distribution in a field is the semivariance function, which is the half of the estimated square difference between sample values at a given distance [38]. The semivariance of parameters was calculated along major directions. The semivariances were not significantly different along major directions, therefore, the isotropic variogram was for each soil property (Z) calculated using following equation;

$$\gamma (h)=\frac{1}{2\;N(h)}+\sum_{i=1}^{N(h)}[z(x_{i}+h)-z{\left(x_{i})\right]}^{2}$$ Eq 6

In the equation, γ(h) is the semivariance of a parameter at lag distance h, N(h) is the number of observation pairs within the lag distance h. The z (xi) and z (xi + h) are the measurements of the parameter at two different points separated by a lag distance h [39]. All pairs separated by a different lag distances were used to calculate the experimental variogram. The variogram models were fitted for each calculated experimental variogram to obtain model parameters, nugget, range and sill. The theoretical semivariogram models were used to predict the values of parameters at unsampled locations.

Spatial distribution maps of parameters were prepared using ordinary kriging interpolation technique. The spatial analyses were carried out using GS+ (Version 7.0) and the maps were produced using ArcGIS 9.2 software in the Geostatistical Analyst 9.2 extension. Ordinary kriging, which is one of the most basic geostatistical interpolation methods under the assumption of intrinsic stationarity for all variables, was utilized as the interpolation method, as the ordinary kriging minimizes the influence of outliers on prediction performance. The ordinary kriging method also estimates the value of the parameters at unsampled location as a linear combination of neighboring observations of all variables [40]. The best fit model was selected based on lowest RMSE and r^2^ values. The validation of maps were carried out using leave-one out cross validation procedure using mean estimation error (MEE) and root mean square estimation error (RMSEE) indices. In cross validation procedure, one observation at a time was temporally removed from the data set and re-estimated from the remaining data. The MEE value provides information on the bias and the RMSEE value is used to assess the prediction accuracy. If the estimation is unbiased and accurate, than the MEE should be close to zero, whereas MSSE close to one [41].

The spatial dependence for all parameters was calculated by the ratio of nugget to sill [42]. The variable is considered having strong spatial dependence when the ratio is less than 25%; the variable is considered having moderate spatial dependence when the ratio is between 25% and 75%; and the variable is considered having a weak spatial dependence when the ratio is greater than 75%.

Descriptive statistics, i.e. the mean value, standard deviation, coefficient of variation (CV), and the maximum and minimum values of soil properties have been determined using SPSS 21 statistical software (SPSS Inc., USA) to describe the central trend and spread of the soil properties datasets. Normality of the data set was examined by Kolmogorov Smirnov test to check the distribution of the variables. The variables without normal distribution were subjected to log transformation. The coefficient of variation (CV) is mainly used to assess the variability of the different data sets. Wilding [43] classified the variability based on the CV values of soil properties, as the CV values of 0–15, 16–35 and > 36% indicate low (least), moderate and high variability, respectively.

## Results and discussion

### Soil salinity and sodicity in the study area

The descriptive statistics for some of soil properties in all soil layers are given in Tables 2 and 3. Soil salinity and sodicity indicated by electrical conductivity (EC) and sodium adsorption ratio (SAR) values indicated high salinity and sodicity with strong variability in each soil layer (Table 3). Clay content in soil layers ranged from 31.5 (90–120 cm) to 63.3% (0–30 cm), and decreased with the increasing the depth of soil profile. Soil particle size distribution has a significant influence on water movement and retention, solute transport in soil, water holding capacity, crop productivity and soil erosion. High clay content slows the movement of water, prevents deep drainage and potentially causes water to accumulate in the profile. Salt accumulation in soil profile is mainly related to the capillary rise of salts from the groundwater table towards the soil surface. Capillary rise in arid regions is the major process for soil salinity that depends both depth of groundwater table and the hydraulic properties of soil profile [44]. Akramkhanov et al. [22] indicated that salinity of sandy soils was lower compared to salinity of finer textured soils. The researchers attributed this to the greater capillary rise of water in finer textured soils compared to the capillary rise in coarse textured soils. Low slope, low precipitation and high capillary rise are the major causes of soil salinity in the study area.

**Table 2:** Particle size distribution of soil samples in the study area.

Unit	Minimum	Maximum	Mean	Std. Deviation	CV (%)
**0–30 cm**					
**Sand %**	5.2	21.6	12.1	4.17	34.39
**Silt %**	27.4	51.8	39.5	5.16	13.05
**Clay %**	39.6	63.3	48.3	5.02	10.38
**30–60 cm**					
**Sand %**	7.5	23.2	14.5	4.15	28.57
**Silt %**	27.3	52.4	39.9	4.70	11.79
**Clay %**	35.9	61.0	45.6	4.82	10.58
**60–90 cm**					
**Sand %**	18.1	23.8	22.1	1.03	4.66
**Silt %**	24.4	44.6	36.5	3.62	9.90
**Clay %**	33.3	54.3	41.3	3.82	9.24
**90–120 cm**					
**Sand %**	20.5	24.4	23.6	0.62	2.61
**Silt %**	24.4	44.7	36.8	3.77	10.24
**Clay %**	31.5	52.5	39.7	3.87	9.76

**Table 3:** Mean soil reaction, electrical conductivity and calcium carbonate content in the study area.

Depth (cm)	Unit	Minimum	Maximum	Mean	Std. Deviation	CV (%)
pH						
**0–30**		7.33	9.02	8.49	0.37	4.40
**30–60**		7.20	9.22	8.62	0.50	5.78
**60–90**		7.56	9.46	8.84	0.38	4.33
**90–120**		7.24	9.41	8.89	0.40	4.45
Electrical Conductivity (EC)						
**0–30**	dS m^-1^	3.00	35.5	11.0	7.14	65.17
**30–60**		3.30	20.48	10.26	3.92	38.20
**60–90**		0.79	19.66	9.35	3.02	32.29
**90–120**		5.38	16.80	9.76	2.30	23.56
Sodium Adsorption Ratio (SAR)						
**0–30**		3.60	57.70	15.96	10.36	64.94
**30–60**		2.70	85.40	25.54	14.96	58.57
**60–90**		11.80	73.50	37.51	15.00	40.00
**90–120**		12.40	89.20	39.64	19.39	48.93
Calcium Carbonate (CaCO_3_)						
**0–30**	%	11.9	27.5	21.9	3.24	14.78
**30–60**		14.5	30.3	21.8	3.57	16.39
**60–90**		13.0	28.0	21.9	3.20	14.62
**90–120**		13.5	29.9	22.9	3.04	13.25

The coefficient of variation (CV) parameter is used to indicate the spatial variability of soil properties [43,45–47]. The variability of all three fractions of particle sizes (except sand in 0–60 cm depth) was very low, that implies a considerable low variation across the study area (Table 2). As indicated by the coefficient of variation, the largest variations in the study area were exhibited by the EC values in 0–30 and 30–60 cm depths and SAR values in all four soil layers (Table 3). The high CV values of soil salinity (EC) and SAR indicated that there is a strong degree of variation within the field. Soil pH exhibited the least variability in study area. The CV of calcium carbonate content also indicated low variability (Table 3). Similar to variability of salinity and SAR values in the study area, the characterization of spatial distribution of soil salinity in two different locations of Khorezm Province, Uzbekistan showed that topsoil salinity was highly variable [22]. High coefficients of variation indicating strong variability in soil salinity have also been reported in the Ili River Valley, China (CV; 71.25%) by Xu et al. [48] in Bohai Sea coastal wetlands, China (CV; 195%) by Lv et al. [18] in the west of Inner Mongolia (CVs for different soil layers was over 100%) by Ren et al. [19] etc.

Soil salinity and sodicity in some parts of the field were very high that cause adverse effects on the growth of crop species. Mean values of pH, EC, SAR and calcium carbonate in surface layer were 8.49, 11.0 dS m^-1^, 15.96 and 21.97%, respectively. Many crops cannot be grown at this salt content. Salinity was at the highest level in soil surface (mean EC: 11 dS m^-1^), and slightly decreased with soil depth. In contrast to salinity, the highest mean SAR value was recorded in 90–120 cm depth, and the SAR values in surface layer were lower compared to subsurface layers. Soil pH values followed a similar trend with SAR values, with minimum mean values determined in surface soils and the pH value increased with soil depth. Mean pH values ranged from 8.49 (0–30 cm) to 8.89 (90–120 cm), and increased with increasing soil depth. The pH in of the field is in the range of having adverse effect on the productivity of several crops due to negative impacts of high pH on availability of plant nutrients. The pH values had the lowest variation in all four soil layers (CV between 4.33 and 5.78%) (Table 3). Similar observations about the CV of pH values were made earlier by He et al. [9]. High sodium content as indicated by high SAR values causes destabilization of soil structure, decrease in soil infiltration rate, increased susceptibility to crusting and difficulties in soil tillage, planting and emergence that adversely affect the growth and yield of crops [49].

The heterogeneity of soil EC, pH and SAR values in the study area indicated insufficiency of the conventional reclamation methodologies based on a mean value. Spatially approaches are, therefore, needed to optimize in the reclamation of saline or saline sodic lands.

Calculation of gypsum or Ca requirement to achieve 100% exchange efficiency is the main target in reclamation of sodic soils, however, this approach ignores the contribution of calcium carbonate in the profile and considers no other sources of Ca present in the solution. Because, the calculation accounts for the mass of Na to be exchanged, and considers the concentration of Na, which will be replaced by Ca [50]. The average calcium carbonate content in 0–120 cm depth was around 22%. The CV values of soil layers indicates that the variation of calcium carbonate content is low within the study area, and calcium carbonate homogenously distributed to the study area and within soil profile. Therefore, neither of the assumptions in calculation of gypsum is valid for the study area. High calcium carbonate content is an advantage in reclamation of sodic soils. In this case, elemental sulfur or sulfuric acid to be applied reduces soil pH and increases the dissolution of calcium carbonate. Calcium ions that will be released into the soil solution will replace the sodium on the colloidal surfaces.

### Spatial distribution of soil properties

The normality test revealed that EC and SAR data of some soil layers did not conform to a normal distribution, therefore; log-transformation was applied to the non-normal EC and SAR data. The data were back transformed to the original data to provide approximate estimates [51] (McGrath et al., 2004). Exponential (0–30 cm), Gaussian (30–60 and 60–90 cm) and spherical models (90–120 cm) were fitted to the experimental variograms (Table 4). The nugget/sill (spatial dependence) ratio explains the degree of heterogeneity in a study area induced by random factors and accounts for the total spatial heterogeneity [52]. The nugget/sill ratio of EC values at soil layers indicated strong spatial dependence representing no significant difference between soil layers. The effects of random factors on salinity decreased with increasing soil depths. The topography, which is a very important factor affecting the spatial distribution of salinity, of the study area is almost flat with a slight slope in south-north and west-east directions. In addition to homogeneity in topography, soil texture is also homogenous as indicated by low CV values. Therefore, the strong spatial dependence of EC values in the study area can be attributed to natural factors such as climate, parent material, topography, or soil type [13,53,54].

**Table 4:** Geostatistical analysis results for various parameters determined in the study.

	pH	Electrical Conductivity	Sodium Adsorption Ratio	CaCO_3_
**0–30 cm**				
Theoretical model	Gaussian	Exponential	Exponential	Gaussian
Nugget	0.0366	0.107	0.082	4.48
Sill	0.02722	0.462	0.606	25.63
Range (m)	3910	3750	4596	5353
r^2^	0.99	0.97	0.97	0.98
RSS	4.34E-04	1.75E-03	2.74E-03	1.34
Standard Error	0.12	0.179	0.141	0.142
r^2^	0.58	0.39	0.51	0.52
Pretreatment	No	log	log	No
Spatial Dependence	134.5	23.2	13.5	17.5
**30–60 cm**				
Theoretical model	Exponential	Gaussian	Spherical	Spherical
Nugget	0.0768	7.53	0.2145	2.32
Sill	0.3676	32	0.445	10
Range (m)	5277	3704	2712	1910
r^2^	0.93	0.99	0.96	0.87
RSS	2.10E-03	2.52	1.25E-03	5.99
Standard Error	0.201	0.173	0.224	0.125
r^2^	0.23	0.48	0.253	0.56
Pretreatment	No	No	Log	No
Spatial Dependence	20.9	23.5	48.2	23.2
**60–90 cm**				
Theoretical model	Gaussian	Gaussian	Gaussian	Gaussian
Nugget	0.0545	6.2	139.9	4.91
Sill	0.29	30.7	279.9	23.72
Range (m)	4132	8622	2809	5547
r^2^	0.98	0.93	0.95	0.96
RSS	6.65E-04	3.95	983	0.929
Standard Error	0.165	0.255	0.2	0.163
r^2^	0.376	0.1	0.32	0.443
Pretreatment	No	No	No	No
Spatial Dependence	18.8	20.2	50.0	20.7
**90–120 cm**				
Theoretical model	Spherical	Spherical	Spherical	Exponential
Nugget	0.0247	0.0001	2.29	0.7
Sill	0.1144	0.047	12.12	233.9
Range (m)	2613	355	3208	756
r^2^	0.94	0.51	0.95	0.97
RSS	3.62E-04	2.46E-05	2.2	141
Standard Error	0.147	0.272	0.143	0.165
r^2^	0.47	0.07	0.487	0.45
Pretreatment	No	Log	No	No
Spatial Dependence	21.6	0.2	18.9	0.3

The geostatistical range (A) is the maximum spatial correlation distance reflecting the size of autocorrelation range of variables, which are affected at both observing and sampling scale [3]. The spatial autocorrelation distance of EC values in the study area were 3750, 3704, 8622 and 355 m for 0–30, 30–60, 60–90 and 90–120 cm depths, respectively (Table 4). The values of SAR were auto correlated to a distance of 4596, 2712, 2808 and 3208 m for 0–30, 30–60, 60–90 and 90–120 cm soil depths, respectively. The large range of EC and SAR values showed that soil salinity and sodicity had a spatial correlation within a wide distance range in the study area, which indicated that the sampling distance >4596 m will be sufficient to explore the spatial variability in soil salinity at 0–60 cm depth.

The interpolation map for the distribution of EC values in each soil layer displayed quite similar patterns. The salinization of soils was high in the north, north-east and north-west of the study area, while it was relatively low in the south-west corner (Fig 3). The accumulation of salts in the study area can be attributed to the direction of dominant slope in the study area. There is a two-way slope in the study area; the first direction of the slope is from south (257 m) to north (240 m) and the second one is from west (240 m) to east (248 m). Akramkhanov et al. [22] reported similar spatial trends for measured soil salinity and the elevation that was accounted for the salinization of low slope end of their study area.

**Fig 2:**
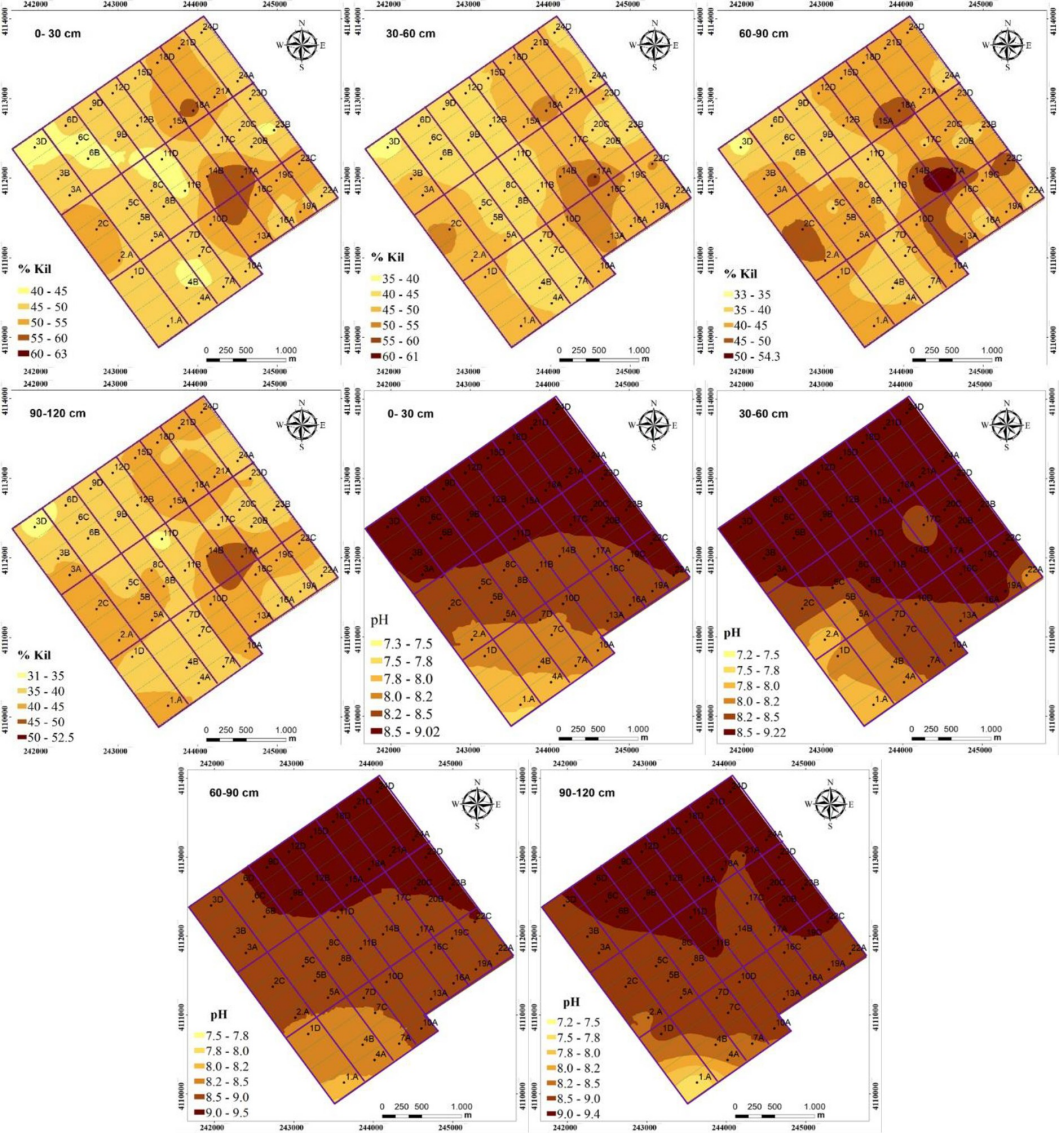
Spatial distribution of clay content and pH values within 0–120 cm of soil profile.

**Fig 3:**
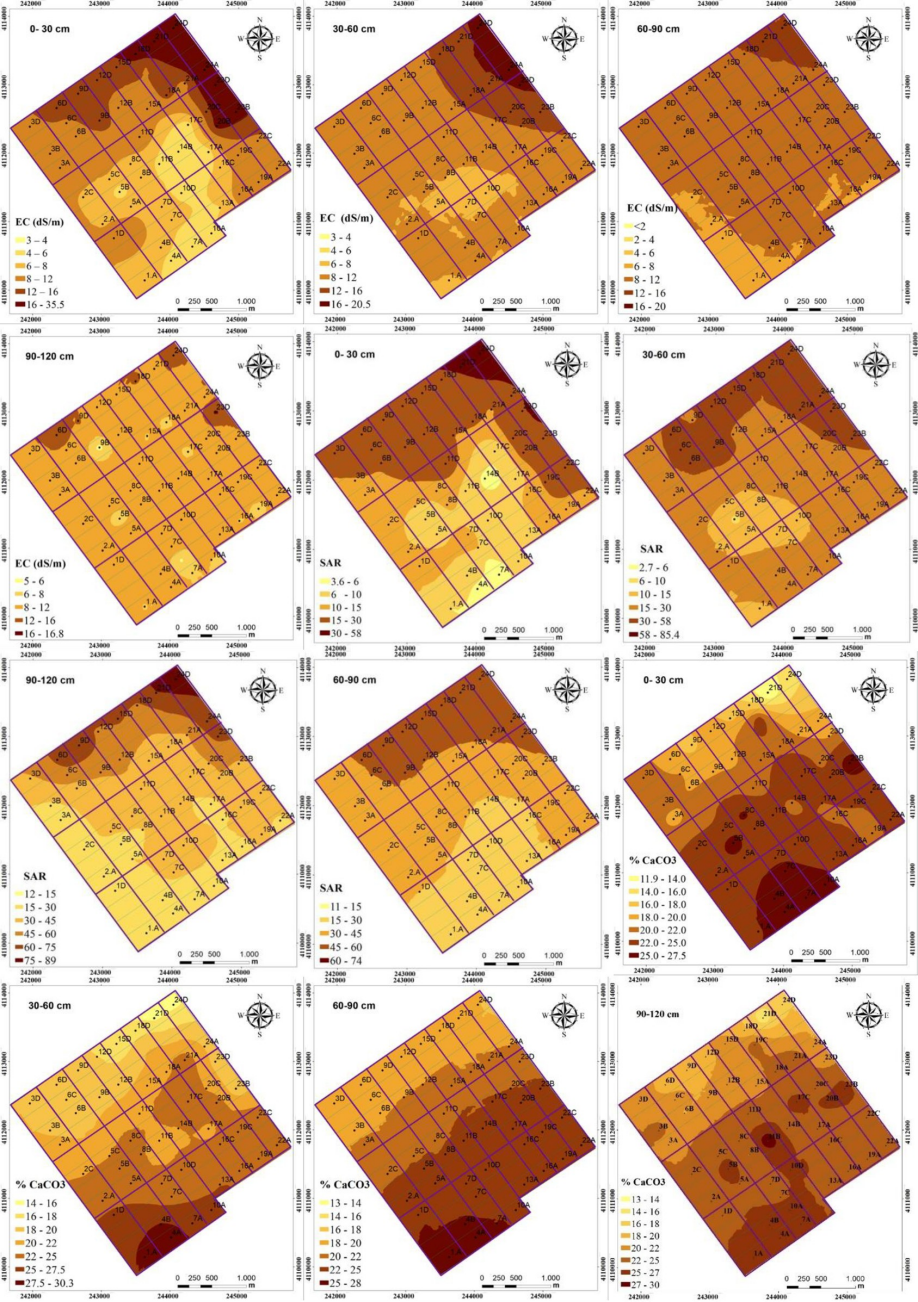
Spatial distribution of electrical conductivity (EC), sodium adsorption (SAR) values and calcium carbonate (CaCO_3_) contents within 0–120 cm of soil profile.

Similar to distributions of soil pH and EC, the high SAR values determined in the north-eastern area of the field (Figs 2 and 3) can be attributed to the influence of slope direction in the study area. This edge of the study area has the lowest elevation and surface runoff has probably occurred through this edge and led to accumulation of salts in soil profile. Yang et al. [55] indicated that soil texture, organic matter, soil water content, subsurface sediments, depth of water table, plant water use, and surface water ponding time and depth as influenced by the microtopography are the major factors affecting the spatial variability of sodicity in a field. In our study area, the northeast edge where the highest SAR, EC and pH values recorded, had the lowest altitude, which induced ponding of surface water and increased the salinity and sodicity.

Soil pH, EC and SAR values displayed significant zonal distributions, gradually decreasing with increasing distance from the north side to south-west corner of the study area (Figs 2 and 3). The study area could be divided into three management zones according to soil pH, EC and SAR values in 0–60 cm depth. The reclamation of the field can be planned based on the 3 distinct areas of high (≥12 dS m^-1^), moderate (12–8 dS m^-1^) and low (<8 dS m^-1^) EC values. The spatial trend analyses of SAR values revealed similar patterns for EC and pH; both gradually decreased from the north to the south-west (Fig 3).

Relative coverage areas of different salinization levels were calculated for different soil layers (Table 5). All soils in the study area showed varying degrees of salinization, with more than 70% in surface more than 90% between 30 and 90 cm depths being strongly saline. In the 0–30 cm layer, 37.9% of the soils were moderately saline, 51.5% was strongly saline and 10.6% was very strongly saline. The salt content increased with depth, and in 30–60 cm depth, 10.2% of soils was moderately saline, while 84.9% was strongly saline, and 4.9% was strongly saline. Majority of salts accumulated in 60–90 cm depth, where soils were either strongly saline (94.1%) or very strongly saline (5.9%). In 90–120 cm depth, soils were slightly saline (4.5%), moderately saline (90.2%) and strongly saline (5.3%). According to soil salinity in the 0–120 cm soil depth, the study area could be divided into three management zones. The management zones in a field are the homogeneous subfield regions that have similar limiting factors or similar attributes, and useful in considering spatial variability of soil properties for adopting precision farming practices [56]. The management zones for soil salinity are moderately saline (4–8 dS m^-1^), strongly saline (8–16 dS m^-1^), and very strongly saline (>16 dS m^-1^). The very strongly saline soils are located on the north and north-east edges of the study area (Fig 3).

**Table 5:** Salinity classes and coverage area of salinity zones in the study area.

EC (dS/m)	Salinity Class*	Area (ha)	Area (%)	Area (ha)	Area (%)	Area (ha)	Area (%)	Area (ha)	Area (%)
** **		0–30 cm		30–60 cm		60–90 cm		90–120 cm	
**3–4**	Slight	0	0	0	0	0	0	0.45	0.1
**4–6**	Moderate	121.8	14.0	0	0	0	0	39.2	4.5
**6–8**	Moderate	208.5	23.9	89	10.2	0	0	**785.5**	**90.2**
**8–12**	Strong	**317.0**	**36.4**	**615.6**	**70.6**	108.8	12.5	45.8	5.3
**12–16**	Strong	131.6	15.1	124.7	14.3	**711.2**	**81.6**	0.11	0
**>16**	Very Strong	92.1	10.6	42.7	4.9	45.8	5.9	0	0
**Total**		871.1	100	871.1	100	871.1	100	871.1	100

*Salinity levels were classified based on Abrol et al. [57].

Relative coverage area of SAR classes based on sodicity hazard were given in Table 6. The accumulation of sodium, as indicated by SAR values, in soil profile followed a contradictory trend with the EC. In the surface, majority of the study area covered slightly sodic soils and 35.6% of the surface soils were light to moderately sodic. The sodium content in soil profiles increased with depth, however, the study area can be divided into four zones based on SAR values (Table 6). The management zones will be none sodic soils (SAR value up to 6), slightly sodic soils (SAR between 6 and ≥15), light to moderately sodic (SAR between >15 and ≥30) and high to very highly sodic soils (SAR value >30). In soil surface, sodicity was not a problem in 3.5% of the study area, 57% had slight sodicity, 35.6% had light to moderate sodicity and 3.9% had high to very sodicity problem (Table 6; Fig 3).

**Table 6:** Sodicity classes for soil surface and coverage area of salinity zones in the study area.

SAR Classes	Sodicity Hazard*	Area (ha)	Area (%)
** **		0–30 cm	
**3–6**	None	30.2	3.5
**6–10**	Slight	230.4	26.4
**10–15**	Slight	266.6	30.6
**15–30**	Light to Moderate	210.2	35.6
**30–58**	High to very High	33.7	3.9
**Total**		871.1	100

* Sodicity hazard classes were determined according to Abrol et al. [57].

**Fig 4:**
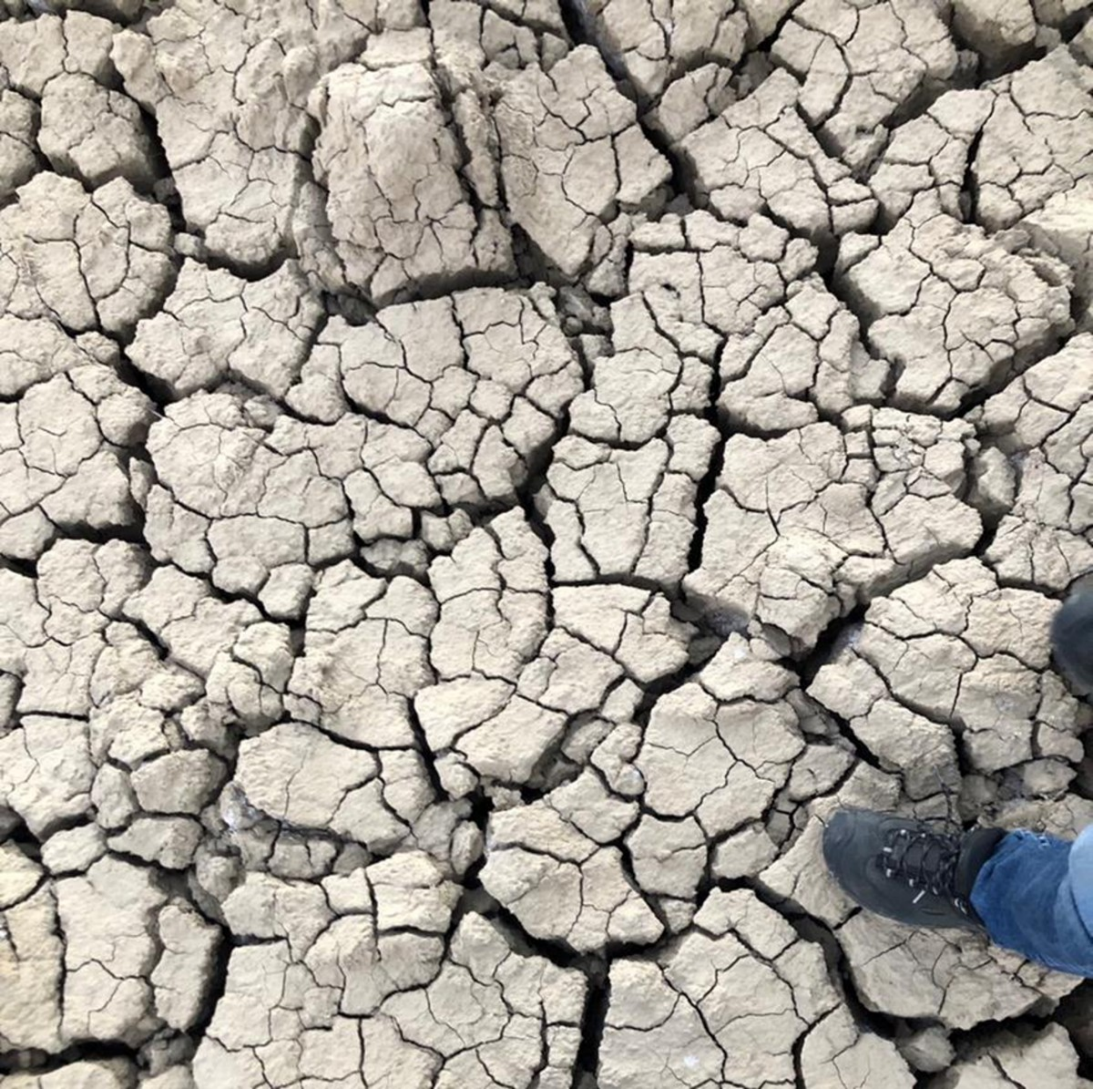
Hard and thick surface crusts on soil surface.

The soils in the study area are clayey and poorly drained. Excessive accumulation of salts especially sodium in the soil led to deterioration of soil physical properties (Fig 4). Poor soil physical properties and high salt contents of the saline-sodic soils are the major constrain limiting the agricultural production in the area. Soil pores are responsible from fluid transport in soil profile, therefore, soil porosity is the most fundamental soil property affecting soil hydraulic conductivity [58], and thus, needs to be improved by the application of chemicals to remove excessive sodium on colloidal surfaces.

### Site specific salinity management in reclamation of soil salinity and sodicity

**Fig 5:**
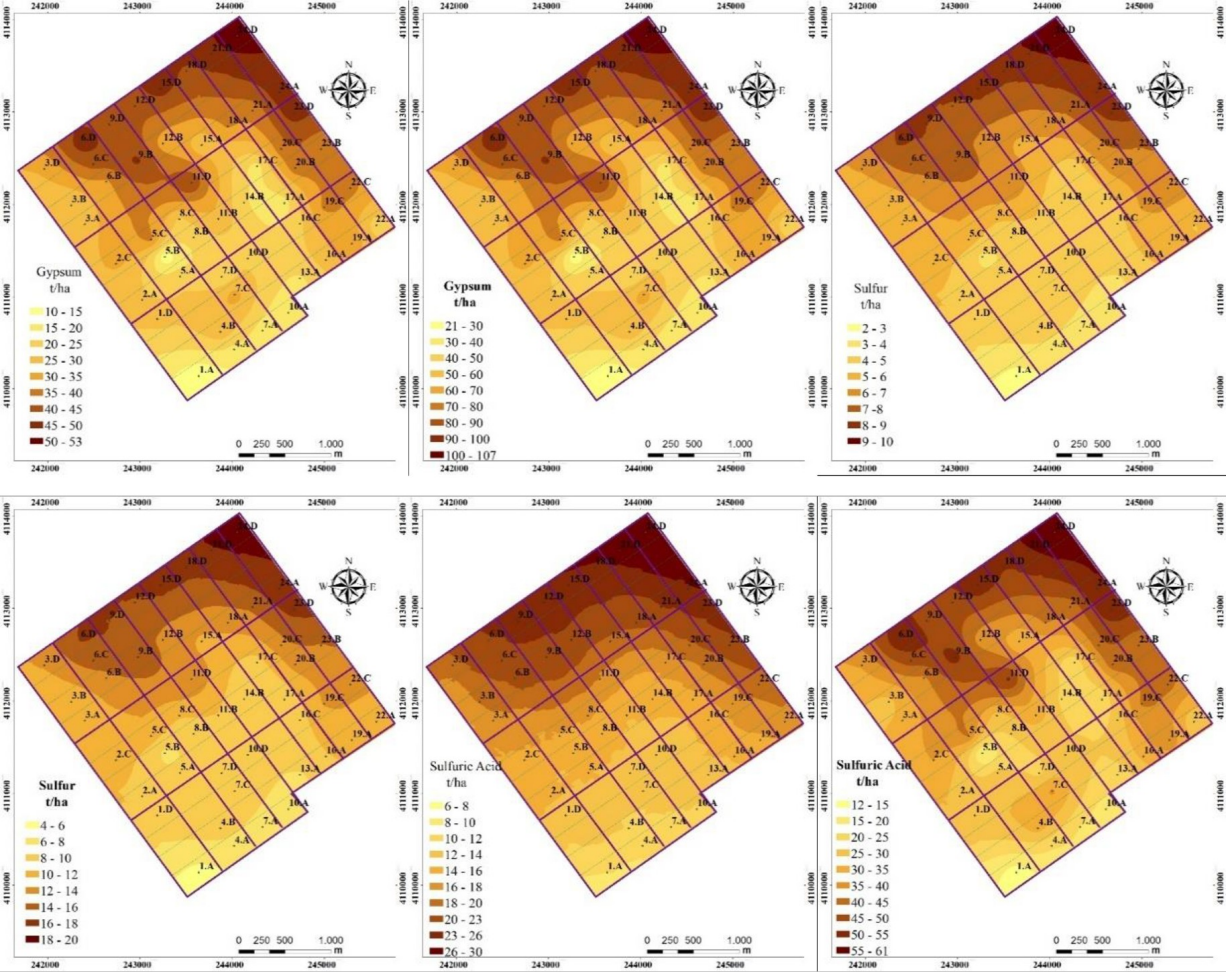
Spatial distribution maps for the amount of gypsum, elemental sulfur and sulfuric acid calculated for 60 and 120 cm depth of soils profile.

The amounts of elemental sulfur, sulfuric acid and gypsum in consideration a required final value of exchangeable sodium percent (ESP_f_ in Eq 1) of 6% within 60 cm and 120 cm of the soil profile are shown in Fig 5. The amount of sulfuric acid, elemental sulfur and gypsum needed to reclaim 120 cm soil profile changes between 12.0–60.8, 3.9–19.9 and 106.9–21.1 ton ha^-1^, while the mean values are between 35.6, 11.7 and 62.6 tons ha^-1^, respectively (Fig 5). If the reclamation is going to cover only 60 cm of soil profile, the amount of sulfuric acid, elemental sulfur and gypsum will be between 6.0–30.4, 2.0–9.9 and 10.6–53.4, while the mean values are 5.82 and 31.30 tons ha^-1^, respectively (Fig 5). Application of chemicals based on one value average calculated for the whole study area as if it is homogenous without accounting for the spatial variability may cause higher or lower of application of chemicals. Lower doses will not be sufficient to replace excessive sodium from exchangeable sites, and excessive doses will cause to spend extra budget. Therefore, the information on soil spatial variability is needed to optimize the use of chemicals with the target of maximizing the remediation process, to make better management decisions aimed at improving the productivity and to minimize the costs while reducing environmental impact. Samra et al. [7] stated that uniform application of chemical amendments for reclamation on the basis of a mean values and neglecting spatial pattern of soil sodicity caused to uneven reclamation of sodicity in the field even after 12 years of crop production. The researchers suggested to adopt spatially sensitive approaches to optimize the reclamation of saline and sodic soils.

**Fig 6:**
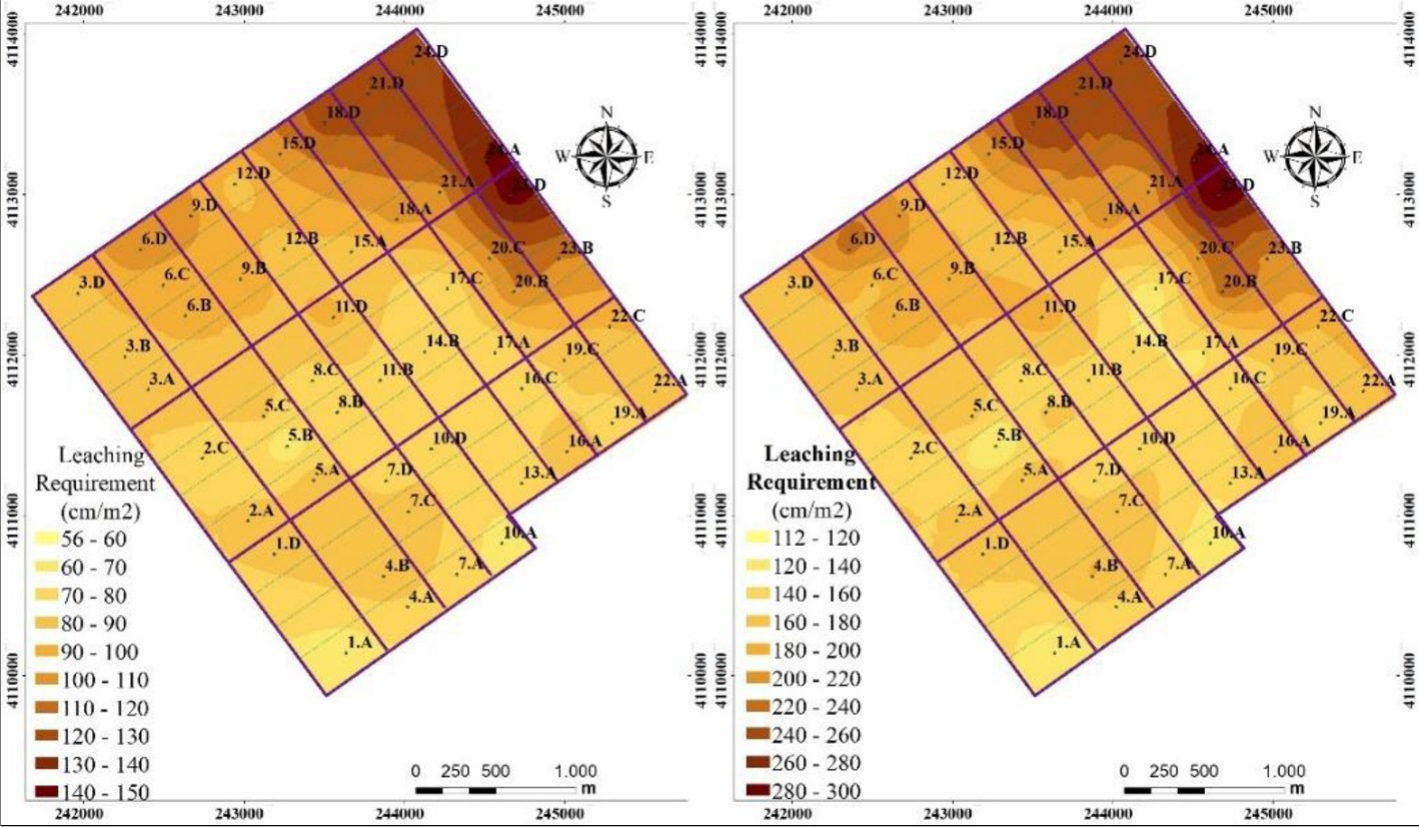
Spatial distribution maps for the amount of leaching water calculated for 60 and 120 cm depth of soils profile.

The leaching of salts from soil profile was projected based on the salt tolerance of the crops to be grown, therefore, the final EC value was set to 2 dS m^-1^ in calculating the amount of leaching water. The amount of water needed to leach down salts from 120 and 60 cm of soil profile was calculated between 112.7–300.0 and 56.4–150.0 ton ha^-1^ and the mean values were 179.7 and 89.8 ton ha^-1^ for 120 and 60 cm soil depths, respectively (Fig 6). In both cases, application of leaching water based on mean values will result in higher or lower leaching water to most locations (Fig 6). Site specific management of soil salinity and sodicity is best handled with knowledge of the spatial distribution of salinity and sodicity in a field. Ideally, water conservation on remediation of agricultural lands is best achieved by applying leaching water where and in the amounts needed to adequately leach salts and to meet the water needed to leach out salts from soil profile. This can be achieved by site-specific application of leaching water, which accounts for within-field variation of water content and salinity. Shaddad et al. [14] showed that 1931 m^3^ of water was saved by the application of site specific management approach to leach salts from a saline soil in 3.1 ha land in Egypt. The net return of saved water was calculated as 12.5 US$ ha^-1^ indicating that the site specific application of leaching water is cost effective. In addition to the economic benefits, site specific leaching is desired for reducing the transport of chemicals that degrade groundwater quality and provides for a more efficient use of limited water supplies [59].

## Conclusion

The results are an important theoretical basis for the improvement and sustainable utilization of saline and saline sodic soils. The distribution of salinity and sodicity parameters I the study area indicated that both salinity and sodicity had a spatially structured phenomena. Therefore, conventional reclamation methodologies based on a mean value of a field assuming independent statistical distributions of input parameters regardless of their spatial considerations may result in uneven reclamation of salinity and sodicity. In addition, leaching and chemical requirements will significantly be reduced when electrical conductivity and sodium adsorption ratio were assumed spatially variable. Moreover, uniform application of chemical or leaching water throughout the field may cause an over application in relatively less sodic or saline locations at the cost of relatively more sodic or saline spots. Hereof, homogenous management zones in a field based on soil properties may improve the efficiency of remediation process and increase economic return by saving water and chemicals used in remediation of saline and sodic soils.
